# Brain Regional Predictors of Surgical Response Are Dynamically Associated with Post-Surgical Pain Relief in Trigeminal Neuralgia †

**DOI:** 10.3390/brainsci16090966

**Published:** 2026-09-13

**Authors:** Emili Adhamidhis, Patcharaporn Srisaikaew, Jerry Li, Timur H. Latypov, Alborz Noorani, Mojgan Hodaie

**Affiliations:** 1 Michael G. DeGroote School of Medicine, McMaster University, Hamilton, ON L8N 3Z5, Canada; emili.adhamidhis@medportal.ca; 2 Division of Brain, Imaging & Behaviour, Krembil Brain Institute, Toronto Western Hospital, University Health Network, Toronto, ON M5G 2C4, Canada; 3 Institute of Medical Science, Temerty Faculty of Medicine, University of Toronto, Toronto, ON M5S 3K9, Canada; 4 The Hospital for Sick Children, Toronto, ON M5G 1E8, Canada; 5 Temerty Faculty of Medicine, University of Toronto, Toronto, ON M5S 3K9, Canada; 6 Division of Neurosurgery, Krembil Neuroscience Center, University Health Network, Toronto, ON M5T 0S8, Canada

**Keywords:** trigeminal neuralgia, predictors of response, neuroplasticity

## Abstract

**Highlights:**

**What are the main findings?**

- Brain regions that have been previously highlighted as predictors of surgical pain relief in TN demonstrate post-surgical alterations in cortical thickness.
- Structural changes in key regions, such as the fusiform gyrus, are related to the degree of surgical pain relief achieved.

**What are the implications of the main findings?**

- Neurobiological changes beyond the level of the nerve occur following surgical treatment for trigeminal neuralgia.
- Relief of chronic neuropathic pain is associated with neuroplasticity across default mode, limbic, dorsal attention, and visual networks.

**Abstract:**

**Background/Objectives:** Trigeminal neuralgia (TN) is a severe chronic neuropathic facial pain condition. Although TN can be amenable to surgical treatments, ~25% of patients may have recurrence of pain. Understanding the role of the central nervous system (CNS) gray matter and its impact on surgical treatment effect remains unclear but is of increasing interest. Previous machine learning models identified 13 neuroanatomical regional predictors of TN surgical response. The implications of these regions and how they might relate to clinical status and surgical outcome are not clear. Here, we attempt to further understand CNS alterations in TN by investigating how these potential regional predictors of surgical response may change following surgical treatment, and whether this is related to the degree of pain relief achieved. **Methods:** Retrospective imaging was acquired from 115 surgically naïve TN patients who underwent Gamma Knife Radiosurgery at Toronto Western Hospital. Patients obtained magnetic resonance imaging scans and reported pain intensity scores before and 3–12 months after surgery. Patients were categorized as responders (≥75% pain relief), partial responders (50–74% pain relief), and non-responders (<50% pain relief). **Results**: Eight regions spanning default mode, visual, limbic, and dorsal attention networks had significant changes in cortical thickness. Responders demonstrated post-operative neuroplasticity in a greater number of regions with higher within-group statistical significance than both partial and non-responders. Thickening of the contralateral fusiform gyrus was correlated with the degree of pain reduction. **Conclusions:** Our findings identify key regions impacted by chronic neuropathic pain, which may be associated with pain relief in TN.

## 1. Introduction

Chronic pain poses an important and challenging health crisis, affecting approximately 30% of people worldwide [1,2,3]. Trigeminal neuralgia (TN) is a severe orofacial neuropathic pain condition [4,5], characterized by unilateral, electric-shock-like facial pain. First-line pharmaceutical treatments can be poorly tolerated or insufficient in managing pain, leading many individuals with TN to eventually require surgical intervention [6]. Unlike other chronic pain conditions, TN can be highly amenable to surgical treatment, and patients can expect remarkable levels of improvement in, or complete resolution of their pain. Approximately 20–25% of patients do not respond to treatment. There is currently a paucity of objective tools that can predict whether or not a patient will have post-surgical pain relief. Further exploration of these predictive biomarkers may provide a greater understanding of the impact of surgery and likelihood of pain relief in TN patients and ultimately improve clinical care of TN by further informing patient decision-making. 

Conventional TN pathophysiology focuses on the concept of neurovascular compression. Contributing to our understanding of TN, recent work has highlighted CNS associations with the experience of pain in TN [7,8,9,10,11,12,13,14]. In comparison to healthy controls, TN patients have shown decreased cortical thickness of several regions, including the insula [15], anterior cingulate cortex [16], dorsolateral prefrontal cortex [7,10], and the fusiform gyrus [11,17,18]. Abnormally decreased gray matter (GM) volume has also been observed in TN, including in key affective and cognitive regions such as the amygdala and hippocampus [13,14,19]. Although we have seen these neuroanatomical abnormalities in people with TN, the role they may play in TN onset, progression, and surgical response remains poorly understood. 

There is emerging interest in CNS-related neuroanatomical abnormalities in TN, particularly in the search for prognostic biomarkers. Along with significant clinical impact, predictive markers of response can further our understanding of contributors to surgical response in TN and guide the development of novel treatments. We have previously applied machine learning models to determine if pre-surgical cortical thickness measures could predict surgical outcome following treatment with Gamma Knife Radiosurgery (GKRS) [11]. We found that structural abnormalities of 13 regions in default mode, visual, somatomotor, limbic, and dorsal attention networks predicted surgical response with >90% accuracy. As a model, TN provides a valuable opportunity to investigate the brain before and after treatment, particularly as surgical treatment can result in significant pain relief. The study of CNS in TN has helped characterize GM abnormalities of the hippocampus, ventral anterior insula, and diffuse trigeminal root entry zone abnormalities, which reverse following surgical pain relief in TN and normalize after pain relief [15,19]. Deeper exploration of how these predictive regions potentially change following surgical treatment in TN, and how this may be related to post-surgical pain relief, would further our understanding of TN. Here, we aim to further address and understand CNS alterations post-surgery in TN by distinctly investigating how previously identified predictive regions potentially modify following treatment. 

## 2. Materials and Methods

Patients with TN, as defined by the International Classification of Headache Disorders 3rd Edition (13.1.1 Trigeminal Neuralgia), treated locally at Toronto Western Hospital were included in this study. Inclusion criteria included (I) a diagnosis of TN, (II) Gamma Knife Radiosurgery (GKRS) as the first surgical treatment for TN, and (III) clinical visits and magnetic resonance imaging (MRI) scans acquired prior to undergoing surgery and 3–12 months following surgery. As all MRI scans were acquired as part of standard clinical care, follow-up time varied across patients based on their availability. Individuals with a history of other neurological conditions such as brain tumors, multiple sclerosis (ICHD-3 13.1.1.2), substance abuse disorders, other chronic pain disorders, active psychiatric conditions, and use of psychoactive medication were excluded from this study. TN patients were subdivided based on pain laterality (left- or right-sided pain); patients with bilateral pain were excluded from this study. TN patients are considered medically refractory primarily based on a profile of adverse side effects and insufficient alleviation of pain from medication, and remaining at Barrow Neurological Institute (BNI) level IV–V [20].

### 2.1. Ethics

This study was approved by the University Health Network Research Ethics Board. As retrospective patient data were used in this study, active recruitment or participation was not required. All MRI scans were de-identified prior to imaging analysis. 

### 2.2. Image Acquisition 

All T1-weighted MRI images were obtained with a 3T GE Signa HDx MRI scanner fitted with an 8-channel head coil at Toronto Western Hospital with the following parameters: fast-spoiled gradient echo (FSPGR), echo time (TE) = 3.0 ms, repetition time (TR) = 8.0 ms, inversion time (TI) = 450 ms, flip angle = 12°, voxel size = 0.86 mm × 0.86 mm × 1.00 mm, acquisition matrix 256 × 256.

### 2.3. Selection of Regions of Interest 

Regions of interest (ROI) were identified from Hung et al. (2021) [11] a priori, which serves as the prelude for the present study. Hung et al. (2021) [11] created a support vector machine (SVM) model, using sequential backward selection (SBS) for feature selection. SBS began with assessing all available features of 68 cortical regions from the Desikan–Kalyanpur–Cohen Atlas and then removing each one sequentially until the desired performance threshold was met [21]. The authors defined the optimal SVM method as the one with the highest cross-validated accuracy and lowest number of features. 

The model chosen was based on cortical thickness of 13 regions that were able to prognosticate TN patients as responders (≥75% pain relief) or non-responders (<75% pain relief) with 90.5% accuracy. These regions are [11] contralateral isthmus cingulate gyrus (ICG), ipsilateral fusiform gyrus (FG), ipsilateral inferior temporal gyrus (ITG), contralateral pars orbitalis (POR), contralateral fusiform gyrus (FG), ipsilateral insula (IN), ipsilateral superior parietal gyrus (SPG), contralateral lateral orbitofrontal gyrus (LOFG), ipsilateral pars triangularis (PTR), contralateral superior temporal gyrus (STG), contralateral lateral occipital gyrus (LOG), contralateral entorhinal cortex (EC), and ipsilateral lateral occipital gyrus (LOG). It is important to note that, rather than being described as left or right, the ROIs in these studies are classified as either contralateral or ipsilateral to the side of TN pain. 

### 2.4. Surgical Interventions and Treatment Response 

All TN patients studied were medically refractory and underwent Gamma Knife Radiosurgery (GKRS) as their first surgical intervention for TN. Patients were treated with either Elekta 4C or Elekta Perfexion systems. The symptomatic trigeminal nerve (ipsilateral to the side of pain) was irradiated with 80 Gy to the 100% isodose line. To minimize the risk of adverse effects, the brainstem dose was restricted to 15 Gy/mm^3^. Follow-up occurred 3–12 months post-surgery.

Prior to and following surgical intervention, TN patients reported their pain intensity on an 11-point Numerical Rating Scale, from 0, indicating complete absence of pain, to 10, defined as the worst pain imaginable. Patients were also rated on the Barrow Neurological Institute (BNI) pain intensity score for neuropathic pain (I: no pain and no medication, II: occasional pain and no medication, III: some pain controlled adequately with medication, IV: some pain not controlled by medication, and V: severe pain with no relief). 

Surgical responders were defined as having ≥75% pain relief and a BNI score of I–III. Non-responders had less than half of their pain resolved (<50% pain relief) with a BNI score of III–V. Individuals who fell in the middle of these classifications with 50–74% pain reduction and a BNI score of II–IV were classified as partial responders.

### 2.5. Cortical Thickness Measurements 

FreeSurfer v7.2.1 was used to extract cortical thickness measurements of all regions of interest using the “recon-all” command. Cortical thickness can be affected by head size [22], and so head size variation was corrected for via the residual method [23], using the following formula:

VOI_adj_ = VOI − b (SGV − SGV_mean_)

where VOI_adj_ is the adjusted volume of interest, VOI is the volume of interest as outputted by FreeSurfer, SGV is the subcortical gray matter volume, b is the slope of the linear regression between VOI and SGV, and SGV_mean_ is the sample mean of the subcortical gray matter volume.

### 2.6. Statistical Analyses 

All statistical analyses were performed in Python v3.12.1. The Shapiro–Wilk normality test was conducted, which revealed that the cortical thickness measurements were not normally distributed (*p*-value < 0.05). Within-group comparisons of cortical thickness before and after surgery were thus conducted with the non-parametric Wilcoxon signed-rank test. Between-group comparisons of response groups were conducted with the Mann–Whitney U Test. All statistical analyses underwent correction for multiple comparisons through False Discovery Rate (FDR) correction, with statistical significance set at *p*-value < 0.05. FDR corrections were applied for each within-group and between-group comparison as well as for correlation analyses individually. As such, all reported *p*-values in this manuscript are FDR-corrected.

## 3. Results

### 3.1. Subject Demographics 

**Figure A1:**
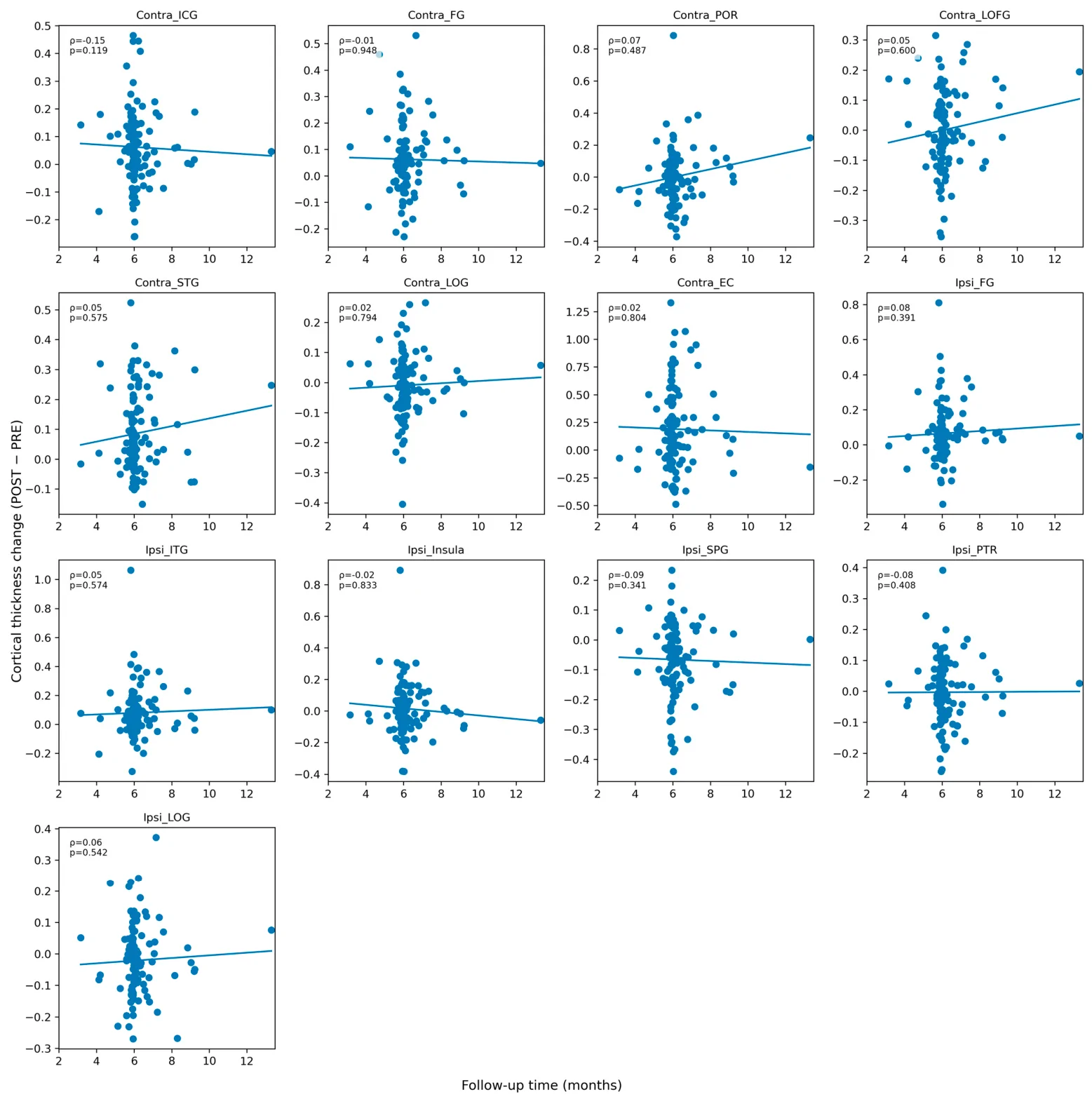
Change in cortical thickness is not significantly correlated with follow-up time in TN patients.

A total of 115 patients with classical TN were included in this study (42M:73F), with an average age at the time of surgery of 65.6 ± 13.1 years. Mean follow-up time was 6.23 ± 1.1 months. Although follow-up time ranged from 3 to 12 months, the majority of patients fell in the 5 to 8-month range, with only one patient having a follow-up time of 3 months (Figure A1). Dataset demographics are available in Table 1, and detailed demographics of each patient are available in Table A1.

A total of 70 patients (24M:46F) were classified as responders and had an average age of 65.4 ± 12.2 years (Table 2). Responders had an average reduction in pain of 96.7%. A total of 59/70 (84%) of the responder cohort had 100% pain reduction. Twenty-two patients (10M:12F) were classified as partial responders and twenty-three patients (8M:15F) were classified as non-responders. Partial responders were comparatively the oldest cohort, with an average age of 69.6 ± 13.2 years, and had the longest pain duration. 

On average, partial responders had a 60.6% reduction in pain (min: 50%, max: 70%, median: 60%) following surgery. Non-responders typically had some degree of pain relief, with an average of 12.3% reduction in pain. Four non-responders reported a post-surgical increase in TN pain (negative values of pain reduction), and four had no change in pain. Of the remaining 15 non-responders that reported some degree of pain relief, the average was 27.1% pain relief (min: 10%, max: 43%, median: 30%). All three groups had similar distributions of pain laterality, with a slight predominance of right-sided pain, as is the general trend in TN [6,24,25]. The demographics and pain characteristics of each response group are available in Table 2.

### 3.2. Sex Distribution 

TN disproportionately affects more female patients than male [26], and this is reflected in our overall dataset, responder cohort, and non-responder cohort, all of which have almost double the number of females than males. Interestingly, this is not seen in the partial responder group, which has a similar sex distribution. There were no significant sex differences in cortical thickness change across response groups (Table A2).

### 3.3. Eight Regions Had Significant Post-Surgical Alterations in Cortical Thickness

Cortical thickness measures of 13 regions that had been previously identified as potential predictors of surgical response were investigated and compared before and after surgery. There were no significant differences between response groups for pre-surgical cortical thickness, post-surgical cortical thickness, or change in cortical thickness (adj. *p*-value > 0.05). However, there were significant within-group differences comparing pre-surgical cortical thickness and post-surgical cortical thickness for 8 regions (Table 3).

None of these changes in cortical thickness were significantly correlated with follow-up time in all TN patients (Figure A1) or within each response group (Table 4).

**Figure 1:**
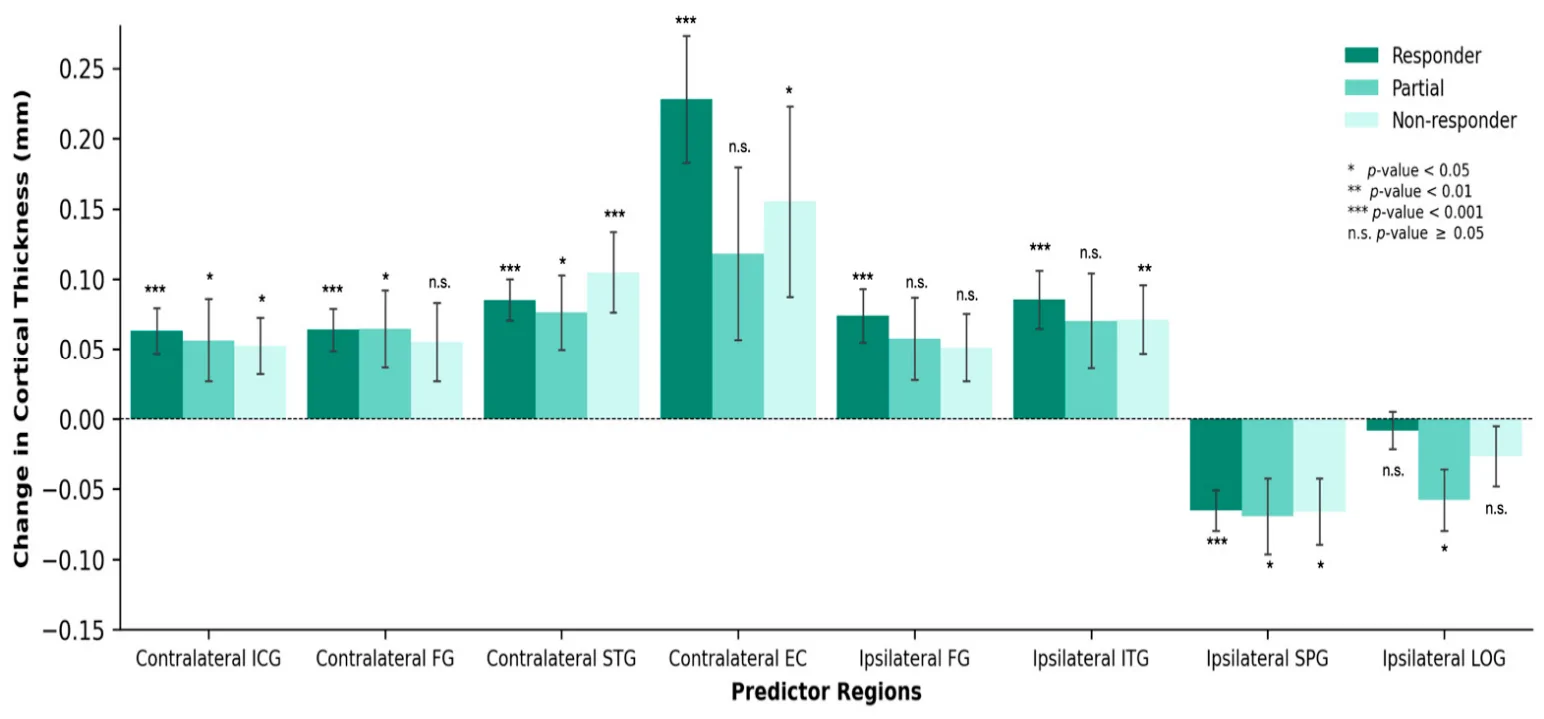
Responders show widespread and significant alterations in cortical thickness following GKRS treatment. Significant post-surgical alterations in cortical thickness alterations of eight regional predictors. Each bar represents the within-group change in pre- to post-surgical thickness; the stars indicate the significance of this change. Responders demonstrated post-operative neuroplasticity in a greater number of regions with higher within-group statistical significance than both partial and non-responders. Error bars depict standard error. Abbreviations: ICG: Isthmus cingulate gyrus, FG: Fusiform gyrus, ITG: Inferior temporal gyrus, SPG: Superior parietal gyrus, STG: Superior temporal gyrus, EC: Entorhinal cortex, LOG: Lateral occipital gyrus.

**Figure A2:**
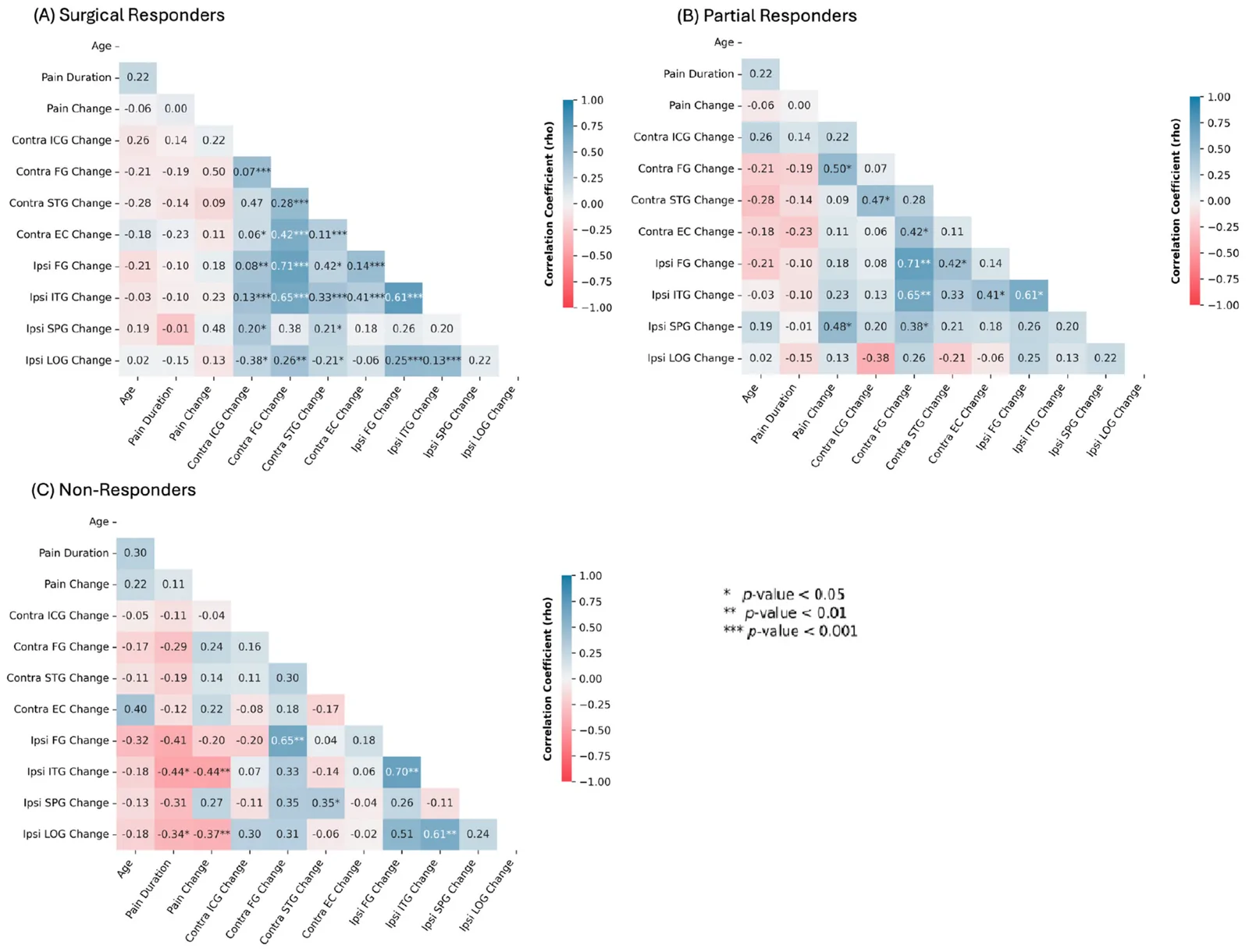
Correlation matrices of (**A**) Responders, (**B**) Partial Responders, (**C**) Non-Responders. Age is not correlated with change in cortical thickness in any region across response groups.

Responders demonstrated significant change in 7 of 13 regions, compared to partial and non-responders, who each had change in only 5/13 regions (Figure 1). Bilateral fusiform gyri undergo post-surgical cortical thickening only in responders and partial responders. Overall, responders typically had a larger magnitude in cortical thickness change compared to non-responders, with the exception of the contralateral superior temporal gyrus, which showed greater GM thickening in non-responders. The ipsilateral lateral occipital gyrus underwent significant cortical thinning in partial responders only. Across groups, none of these changes were significantly correlated with age (Figure A2). The change in cortical thickness from pre- to post-surgery for each significant region is visualized in Figure 1.

### 3.4. Post-Surgical Network-Specific Patterns in Cortical Thickness Alterations 

**Figure 2:**
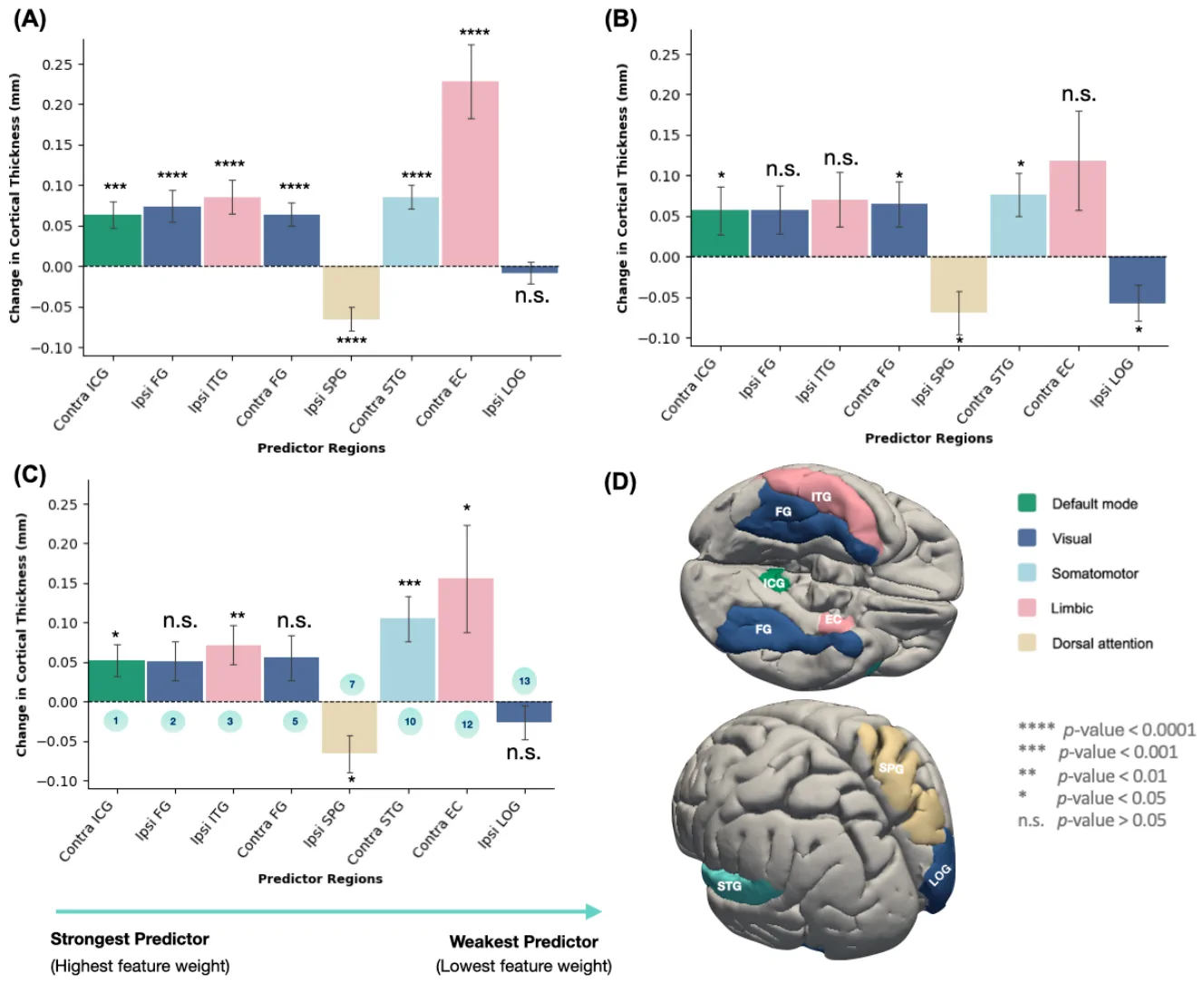
Networks and relative predictor strength of key regions. Changes in cortical thickness in (**A**) Surgical responders, (**B**) Partial responders, and (**C**) Non-responders. Predictor regions are organized on the x-axis from strongest to weakest predictor, as identified by Hung et al. (2021) [11], numbered with their relative order of greatest feature weight. (**D**) Visualizes the significant brain regions and color-codes them according to functional network. Abbreviations: Contra: Contralateral, Ipsi: Ipsilateral, ICG: Isthmus cingulate gyrus, FG: Fusiform gyrus, ITG: Inferior temporal gyrus, SPG: Superior parietal gyrus, STG: Superior temporal gyrus, EC: Entorhinal cortex, LOG: Lateral occipital gyrus.

Regions in the default mode, visual, somatomotor, limbic, and dorsal attention networks demonstrated post-surgical changes in cortical thickness. Interestingly, the visual network showed treatment-specific modifications, with significant changes in these regions only being seen in responders and partial responders, and none at all in non-responders (Figure 2). A key region in the visual network is the fusiform gyrus, which only demonstrates significant cortical thickening in responders and partial responders. 

Each region was ranked by feature weight, reflecting its relative importance to the overall accuracy of the preceding study’s predictive model [11]. As such, a region with a greater feature weight is considered to be a relatively stronger predictor region. Figure 2 shows the regions that have undergone significant post-surgical change in order from strongest to weakest predictive regions. Four out of the top five strongest predictor regions underwent significant post-surgical cortical thickening in surgical responders. These are the contralateral isthmus cingulate gyrus (ranking: 1), ipsilateral fusiform gyrus (ranking: 2), ipsilateral inferior temporal gyrus (ranking: 3), and contralateral fusiform gyrus (ranking: 5).

### 3.5. Pain Relief Is Significantly Correlated with Post-Surgical Modifications of the Fusiform Gyrus in Partial Responders 

**Figure 3:**
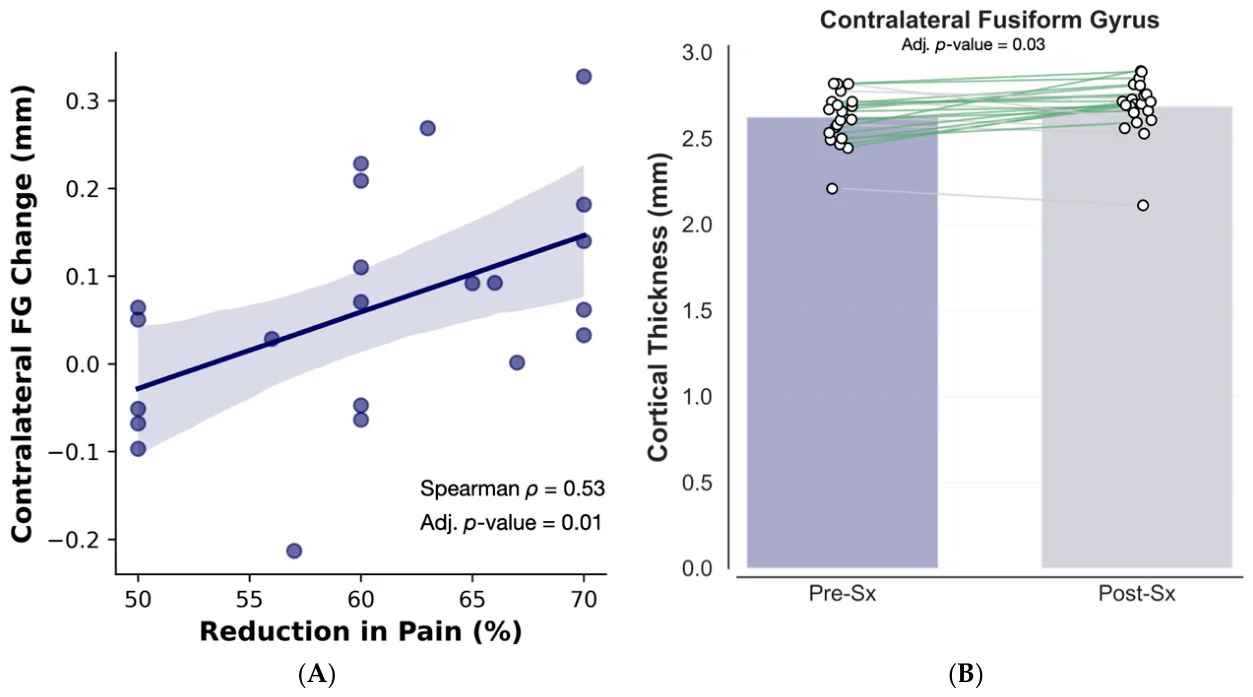
Cortical thickening of the fusiform gyrus is positively correlated with greater pain relief in partial responders. (**A**) Positive correlation between percentage pain relief following treatment and the change in cortical thickness of the contralateral fusiform gyrus of partial responders. (**B**) There is a significant increase in cortical thickness following surgical treatment. Individual cortical thickness points at each time point are shown with blue lines representing decreases in cortical thickness, and green lines representing increases. Abbreviations: FG: fusiform gyrus.

The contralateral fusiform gyrus was the only region found to be significantly associated with pain relief. This was observed in the partial responder group (Figure 3). In this group, cortical thickening of the fusiform gyrus was positively correlated with percent pain reduction post-GKRS treatment.

## 4. Discussion

There is increasing interest in identifying objective markers that relate to the clinical expression of pain. This need is particularly relevant in TN, since, as a surgically treatable condition, there is a dearth of established diagnostic or predictive markers to guide patient selection, prognostication, or treatment discussions. The identification of candidate markers may support more informed clinical decision-making and consent and provide insight into CNS mechanisms underlying pain and associated cognitive and affective features. In this context, structural measures such as cortical thickness permit the detailed assessment of brain regions implicated in pain processing. In the present study, we examined 13 previously identified cortical thickness-based predictors of surgical outcome to determine whether these regions dynamically alter in thickness following treatment and if changes relate to the degree of pain relief achieved. We further sought to characterize these alterations at the network level to identify functional systems modulated by surgical pain relief. We demonstrate post-surgical changes in cortical thickness across regions spanning default mode, limbic, visual, and attention networks, with greater magnitude and extent of change that correlates with greater pain relief. These findings support the presence of multi-network modulatory processes associated with pain chronicity and resolution, involving domains such as emotional memory, attention, and higher-order cognitive processing.

In the present study, we include a partial responder group to capture the broad range of responses to surgery for TN. We identified a significant correlation between cortical thickening of the contralateral fusiform gyrus and degree of pain relief within the partial responder group. This correlation was not observed in complete responders or non-responders, likely due to ceiling and floor effects. The FG, previously linked to pain-related memory, affective imagery, and higher-order visual processing, may represent a dynamic cortical substrate that reflects affective–cognitive contributions to pain persistence and relief. Along with the FG, several of these predictive regions involved in default mode, limbic–paralimbic, and sensory–motor networks have also demonstrated accelerated brain aging in chronic pain patients, including TN [27,28]. Our findings provide evidence of neuroplasticity in TN, dynamic alterations as a result of surgical treatment, and suggest that targeted imaging of select cortical regions may hold potential translational value for post-surgical monitoring and future individualized prognostication. 

### 4.1. Regional Predictors of Treatment Response Undergo Post-Surgical Cortical Modifications 

This study examines whether previously identified neuroanatomical regions that have a role in prediction of surgical outcome also demonstrate morphological changes following surgical treatment. Of the top five strongest regional predictors of response, four underwent significant cortical thickening in surgical responders compared to only two in partial and non-responders. The ICG was the strongest predictor of response and had undergone post-surgical thickening in all response groups, with the greatest and most significant cortical thickening in surgical responders. The ICG is a node of the self-referential default mode network (DMN), and abnormalities in gray matter morphology of this region have been associated with depression [29,30,31]. Other regions of the DMN investigated in this study include the pars orbitalis and triangularis, neither of which displayed significant change in cortical thickness. In TN, abnormalities in DMN connectivity have been observed [7,14,32,33] and are presumed to be related to cognitive components of chronic pain such as rumination [34]. 

**Figure A3:**
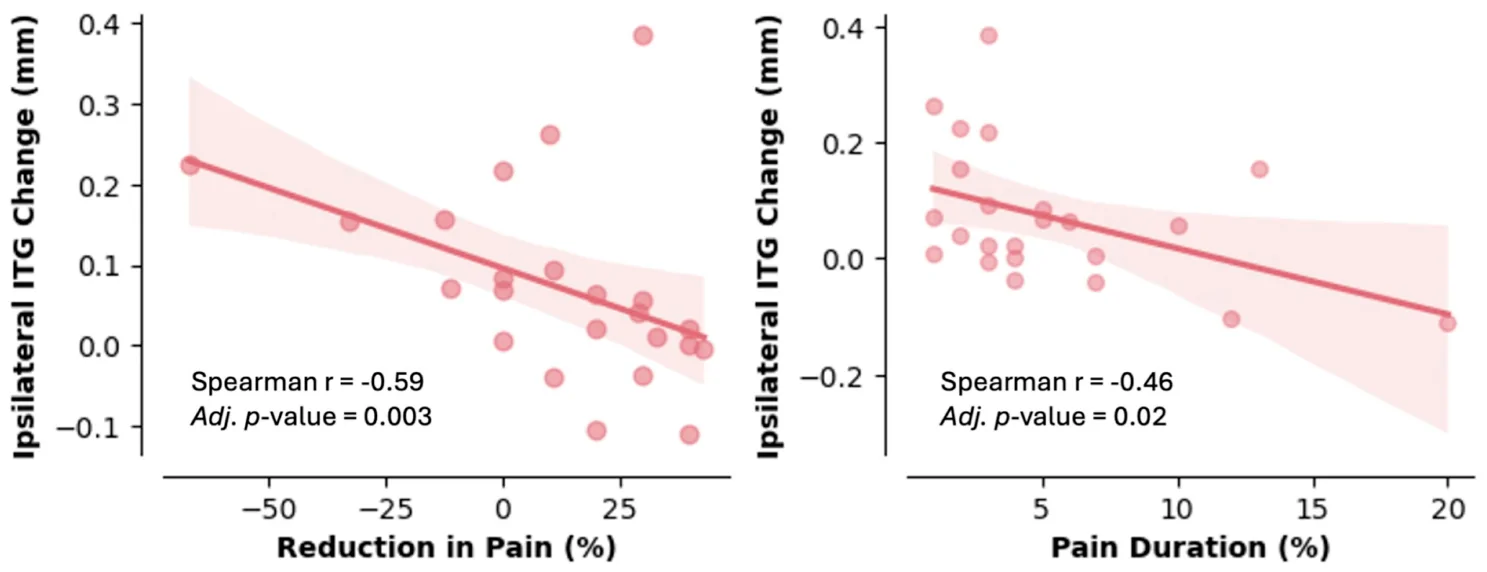
The ipsilateral inferior temporal gyrus in non-responders is correlated with pain relief and duration.

The ipsilateral and contralateral fusiform gyri were the second and fifth strongest predictors of response, respectively, and had significant post-surgical cortical thickening only in patients with at least 50% pain relief. The third strongest predictor, the inferior temporal gyrus, increased in cortical thickness in both responders and non-responders. The ITG has been found to have abnormally decreased pre-surgical cortical thickness in TN and was correlated with both pain severity and duration [7]. The latter relationship was supported in our findings in non-responders only. Cortical thickness change in the ITG in non-responders was significantly negatively correlated with both pain duration (adj. *p*-value = 0.02) and degree of pain relief (Figure A3) (adj. *p*-value = 0.003). As such, duration and severity of pain may affect the composition of the ITG differently in responders versus non-responders. The contralateral STG had greater post-surgical thickening in non-responders compared to responders, a trend that was not seen for any other region. Interestingly, increased cortical thickness of the STG has been associated with post-traumatic stress disorder [35,36] and may reflect emotional distress in TN patients following unsuccessful surgical treatment. 

Impaired memory is a common feature in chronic pain conditions, including TN [37]. We have seen abnormally reduced GMV of the hippocampus in TN, and that following surgical pain relief, hippocampal GMV can increase and normalize to the level of healthy controls [19]. Our findings demonstrate a similar trend, with significant gray matter increases in the entorhinal cortex, another key memory hub via its connections to the hippocampus. Cortical thickness of the EC decreases by 0.19% annually in healthy individuals [38]. We report that in TN surgical responders, cortical thickness of the EC increased by an average of 0.22 mm or roughly 7.7% of its pre-surgical thickness (2.97 mm) (Table A3). Cortical thickness changes in partial and non-responders can be found in Table A4 and Table A5. As the EC is a small region, our results may be confounded by FreeSurfer’s ability to accurately segment the EC. Our findings of EC thickening align with previously published literature demonstrating post-surgical neuroplasticity in key memory and cognition regions in TN and may suggest that cognitive processes can recover following pain relief. 

Several of the neuroanatomical regions investigated in the present study have also been highlighted as regions that contributed to accelerated brain aging in TN, defined as an increased gap between chronological age and MR-based machine-learning predicted age [27,28,39]. Hung et al. (2022) [27] looked at 118 cortical thickness features. Fifteen top features were studied in detail and contributed to their brain-AGE calculation. Our report, studied in a different TN cohort, points to six shared neuroanatomical regions, including the fusiform gyrus, parahippocampal gyrus, cingulate gyrus, insula, superior temporal gyrus, and pars triangularis [27]. Two regions, the fusiform and superior temporal gyri, demonstrated increased cortical thickness following surgical pain relief (Figure 1, Table 3). Accelerated brain aging has been seen across different neurological conditions, including depression, neurodegenerative diseases, and multiple sclerosis [40,41,42,43], and is associated with impaired functional capacity and cognition [44,45,46,47]. Interestingly, TN accelerated brain aging was associated with affective components of pain, greater pain catastrophizing, and pain-related anxiety, but not with pain intensity or disease duration [28]. The regions studied in the present study that are both brain-AGE and surgical response biomarkers in TN, such as the FG and STG, are not classically associated with pain processing but rather multisensory integrative emotional processing [36,48,49,50,51,52,53]. Taken together, this suggests the emotional burden of chronic neuropathic pain as a potential shared, modulatory factor linking accelerated brain aging, neuroplasticity, and surgical response in TN. 

### 4.2. Post-Surgical Thickening of the Fusiform Gyrus Is Correlated with Pain Reduction 

Surgical responders typically have extremely favorable surgical outcomes, often experiencing complete resolution of their pain. Indeed, in the present cohort, 84% (59/70) of surgical responders had a 100% reduction in pain. Because of this, it has been difficult to identify a relationship between pain relief and changes in cortical thickness in this group. With our introduction of a partial response group, which has more gradual reductions in pain, we aimed to evaluate how post-surgical changes in cortical thickness may relate to pain relief. In a novel finding, we report that post-surgical modification of the fusiform gyrus is significantly correlated with the degree of pain relief in TN partial responders. The FG is functionally heterogeneous, with distinct specialized subregions. For instance, the fusiform face area, which is responsive to images of faces, is anatomically and functionally separate from the fusiform body area, which is responsive to images of bodies [54,55]. Through white matter connections to the hippocampus and temporal lobe, the FG is also involved in higher-order cognitive and semantic processes such as object form and manipulation, and word recognition [56,57,58]. Moreover, presumably via its white matter connections with the hippocampus, the FG is implicated in autobiographical memory and shows increased activation when asked to re-experience personal memories and events [59,60]. 

More recently, the FG has been implicated in pain. In healthy controls, activation of the FG has been seen with visualizing themselves undergoing pain and in response to images of pained expressions of others, as well as in autobiographical memory retrieval of painful experiences [61,62]. Both structural and functional abnormalities of the FG have been seen in chronic pain. There is greater resting-state connectivity [63,64,65,66] and increased activation of the FG when visualizing painful experiences in chronic pain patients compared to healthy controls [67]. In TN patients, both reduced cortical thickness and increased resting-state connectivity of the FG have been observed [11,18,68,69]. The structure of the FG was also a key feature in a data-driven TN pain signature that could distinguish multiple sclerosis-TN from multiple sclerosis patients with 93.4% accuracy [70]. In the present study’s prelude by Hung et al. (2021) [11], pre-surgical cortical thickness of the ipsilateral and contralateral FG was the second and fifth strongest predictors of treatment response in TN. Our findings further support a role of the FG in TN pain resolution, and suggest that post-surgical neuroplasticity of the FG may be distinctly involved with surgical pain relief in TN. Though it remains unclear what may be driving changes in the FG in TN, it may involve pain visualization and memory. Anxiety and pain catastrophizing are common in chronic pain patients, and both are associated with less favorable treatment outcomes [71,72]. Studies have shown that chronic pain patients who create mental images of their pain and have greater pain imagery also have greater rates of anxiety, depression, and catastrophizing [73,74]. As such, abnormalities in FG structure and function may relate to the affective and cognitive dimensions of pain, such as increased pain imagery, re-experiencing painful memories, or rumination in TN. 

While several cortical regions identified in this and related studies predict pain outcomes following surgical intervention, not all these regions are directly involved in the sensory or affective encoding of pain [11,75,76,77,78]. Areas such as the precuneus, posterior cingulate, and parahippocampal gyrus are generally associated with memory, self-referential processing, and resting-state network dynamics [79,80,81,82]. That these areas are repeatedly emerging as predictors of outcome may suggest a broader modulatory process that influences pain chronification and recovery. These processes include emotional memory encoding or default mode network regulation. Bushnell and colleagues have emphasized the cognitive–evaluative and emotional dimensions of chronic pain, highlighting how cortical circuits beyond traditional nociceptive areas contribute to pain modulation and experience [77]. Our observations on cortical alterations may serve as markers of systemic adaptations to chronic pain or indicators of vulnerability and resilience, rather than direct correlates of pain intensity. This distinction is critical for interpreting imaging biomarkers of outcome and for understanding the distributed nature of pain processing and recovery.

### 4.3. Limitations 

Our study has limitations to be addressed. Although neuroimaging is an extremely valuable tool in observing brain morphology, it cannot provide information on what cellular mechanisms are occurring and driving the structural changes we observe. As such, we cannot be sure what neurobiological mechanisms may be driving our findings or make conclusions about a causal relationship with pain relief. Similarly, as we have not collected and analyzed cognitive or behavioral questionnaires in these patients, we are unable to conclude which dimension of their pain may be contributing to the changes we see. Future studies should integrate behavioral measures with neuroimaging analysis. TN patients experience severe pain and use anti-convulsant medications such as carbamazepine, oxcarbazepine, and gabapentin to manage their pain. Following surgical treatment with GKRS, patients often remain on pain medication or slowly titrate their dosage. As such, we could not control for the effect of anti-convulsant pain medication on pre- or post-surgical cortical thickness in this cohort. Although previous studies have indicated that anti-convulsant medications may not impact CNS gray matter [83], we cannot completely rule out potential medication effects on our findings. Pain intensity was assessed retrospectively through clinical records and was determined with two scales, the Numerical Rating Scale and BNI, that are given in clinic as standard of care. These scales do not provide information on several dimensions of the patient’s pain experience, including the frequency and duration of TN pain paroxysms and pain unpleasantness. We are thus limited in our analysis of what aspect of their pain may be contributing to the changes in cortical thickness that we see. 

## 5. Conclusions

We report dynamic behavior of the brain following resolution of chronic pain and provide novel evidence of a significant association between post-surgical neuroanatomical changes and the degree of pain relief. Our findings contribute to a growing body of literature implicating a greater role of the CNS in the process of TN pain relief and identify the fusiform gyrus as a potential key region of interest for further exploration in TN.

## Figures and Tables

**Table 1:** Demographic summary of included TN patients.

Dataset Demographics	
Sample size (n)	115
Age (years)	65.6 ± 13.1
Sex (males:females)	42:73
Pain side (left:right)	51:64
Pain duration (years)	5 (IQR 3–10)
Follow-up time (mos)	6.23 ± 1.1
Responder (n)	70 (61%)
Partial responder (n)	22 (19%)
Non-responder (n)	23 (20%)

mean ± SD.

**Table 2:** Demographic and pain characteristics of response groups.

	Responder ≥75% Pain Relief	Partial Responder 50–74% Pain Relief	Non-Responder <50% Pain Relief
Sample size (n)	70	22	23
Age (years)	65.4 ± 12.2	69.6 ± 13.2	62.3 ± 15.1
Sex (males:females)	24:46	10:12	8:15
Pain side (left:right)	31:39	10:12	10:13
Pain duration (years)	7.5 ± 7.3	9.5 ± 8.8	5.2 ± 4.6
Pre-Surgery NRS	9.2 ± 1.0	9 ± 1.1	8.9 ± 1.7
Post-Surgery NRS	0.3 ± 0.7	4 ± 0.8	7.6 ± 1.9
Mean follow-up (mos)	6.35 ± 1.1	5.91 ± 1.0	6.13 ± 0.9
Pre-Surgery BNI			
Group I–III	2	1	2
Group IV–V	68	21	21
Post-Surgery BNI			
Group I–III	70	15	1
Group IV–V	0	7	22
Pain Reduction (%)	96.7 ± 7.5	60.6 ± 7.35	12.3 ± 18.7

**Table 3:** Pre- vs. post-surgical cortical thickness of 13 neuroanatomical predictor regions in TN response groups.

	Responder ≥75% Pain Relief, BNI I–III	Partial Responder 50–74% Pain Relief, BNI III–IV	Non-Responder <50% Pain Relief, BNI IV–V
Regional Predictor	Pre- vs. Post-Sx Change, Mean ± SD (adj *p*-Value < 0.05)	Pre- vs. Post-Sx Change, Mean ± SD (adj *p*-Value < 0.05)	Pre- vs. Post-Sx Change, Mean ± SD (adj *p*-Value < 0.05)
**Contralateral:**			
**Isthmus cingulate gyrus**	**+0.063 ± 0.14 mm**	**+0.056 ± 0.14 mm**	**+0.052 ± 0.09 mm**
**Fusiform gyrus**	**+0.064 ± 0.13 mm**	**+0.064 ± 0.13 mm**	+0.055 ± 0.14 mm
**Pars orbitalis**	+0.013 ± 0.18 mm	+0.017 ± 0.13 mm	−0.036 ± 0.13 mm
**Lateral orbitofrontal gyrus**	−0.003 ± 0.13 mm	−0.018 ± 0.14 mm	+0.041± 0.11 mm
**Superior temporal gyrus**	**+0.085 ± 0.12 mm**	**+0.076 ± 0.12 mm**	**+0.105 ± 0.14 mm**
**Lateral occipital gyrus**	−0.009 ± 0.10 mm	−0. 019 ± 0.11 mm	+0.002 ± 0.09 mm
**Entorhinal cortex**	**+0.228 ± 0.38 mm**	+0.118 ± 0.29 mm	**+0.155 ± 0.32 mm**
**Ipsilateral:**			
**Fusiform gyrus**	**+0.074 ± 0.16 mm**	+0.057 ± 0.14 mm	+0.051± 0.12 mm
**Inferior temporal gyrus**	**+0.085 ± 0.17 mm**	+0.070 ± 0.16 mm	**+0.071± 0.12 mm**
**Insula**	+0.027 ± 0.16 mm	−0.033 ± 0.14 mm	+0.019 ± 0.15 mm
**Superior parietal gyrus**	**−0.065 ± 0.12 mm**	**−0.069 ± 0.13 mm**	**−0.066 ± 0.11 mm**
**Pars triangularis**	0.000 ± 0.1 mm	−0.026 ± 0.11 mm	0.004 ± 0.08 mm
**Lateral occipital gyrus**	−0.008 ± 0.11 mm	**−0.058 ± 0.1 mm**	−0.023 ± 0.1 mm

**Table 4:** No significant correlation between cortical thickness change and follow-up time across response groups.

Region of Interest	Responder	Partial Responder	Non-Responder
	Cortical Thickness Change Correlation with Follow-Up Time Spearman Rho (*p*-Value)	Cortical Thickness Change Correlation with Follow-Up Time Spearman Rho (*p*-Value)	Cortical Thickness Change Correlation with Follow-Up Time Spearman Rho (*p*-Value)
Contralateral:			
Isthmus cingulate gyrus	−0.017 (0.886)	−0.369 (0.091)	−0.298 (0.165)
Fusiform gyrus	0.169 (0.161)	−0.246 (0.269)	−0.262 (0.225)
Pars orbitalis	0.213 (0.077)	0.235 (0.293)	−0.401 (0.058)
Lateral orbitofrontal gyrus	0.151 (0.212)	−0.059 (0.795)	−0.159 (0.466)
Superior temporal gyrus	0.179 (0.138)	−0.330 (0.133)	−0.0004 (0.998)
Lateral occipital gyrus	0.129 (0.286)	0.303 (0.170)	−0.171 (0.435)
Entorhinal cortex	0.013 (0.918)	−0.264 (0.234)	0.108 (0.624)
Ipsilateral:			
Fusiform gyrus	0.105 (0.386)	0.196 (0.381)	−0.065 (0.769)
Inferior temporal gyrus	0.126 (0.297)	−0.098 (0.665)	−0.086 (0.698)
Insula	0.056 (0.644)	−0.193 (0.389)	−0.294 (0.173)
Superior parietal gyrus	−0.046 (0.706)	−0.301 (0.173)	−0.051 (0.816)
Pars triangularis	0.040 (0.744)	−0.282 (0.203)	−0.232 (0.287)
Lateral occipital gyrus	0.004 (0.973)	0.303 (0.170)	−0.066 (0.764)

## Data Availability

Anonymized patient data are available upon reasonable request, due to privacy restrictions.

## Abbreviations

The following abbreviations are used in this manuscript:

TN	Trigeminal neuralgia
CNS	Central nervous system
MRI	Magnetic Resonance Imaging
GKRS	Gamma Knife Radiosurgery
GM	Gray matter
ICHD-3	International Classification of Headache Disorders 3rd Edition
FSPGR	Fast spoiled gradient echo
TE	Echo time
TR	Repetition time
TI	Inversion time
ROI	Region of interest
SVM	Support vector machine
SBS	Sequential backward selection
ICG	Isthmus cingulate gyrus
FG	Fusiform gyrus
ITG	Inferior temporal gyrus
POR	Pars orbitalis
IN	Insula
SPG	Superior parietal gyrus
LOFG	Lateral orbitofrontal gyrus
PTR	Pars triangularis
STG	Superior temporal gyrus
LOG	Lateral occipital gyrus
EC	Entorhinal cortex
BNI	Barrow Neurological Institute
VOI	Volume of Interest
SGV	Subcortical gray matter volume
FDR	False discovery rate
DMN	Default mode network

## Appendix A

**Table A1:** Complete and detailed demographics of TN patients included in this study.

Subject ID	Sex	Age (y)	Pain Side	Pain Duration (y)	Pre-Sx NRS	Post-Sx NRS	% Pain Reduction	Pre-Sx BNI	Post-Sx BNI	Surgical Response
TN01	F	75	R	3	10	0	100	V	I	Responder
TN02	F	71	R	7	8	0	100	IV	I	Responder
TN03	F	58	R	3	10	0	100	IV	I	Responder
TN04	F	71	L	5	10	2	80	V	III	Responder
TN05	F	57	L	4	10	0	100	IV	III	Responder
TN06	M	85	R	7	5	0	100	IV	I	Responder
TN07	F	70	L	10	10	2	80	V	III	Responder
TN08	M	64	L	3	7	0	100	V	I	Responder
TN09	M	72	R	12	10	0	100	V	I	Responder
TN10	F	67	L	2	9	0	100	V	III	Responder
TN11	M	62	R	9	8	0	100	IV	I	Responder
TN12	F	79	R	3	10	0	100	V	III	Responder
TN13	F	71	L	15	8	0	100	IV	III	Responder
TN14	F	63	R	10	8	0	100	IV	II	Responder
TN15	F	69	R	1	8	0	100	IV	II	Responder
TN16	M	73	R	40	10	0	100	IV	III	Responder
TN17	F	50	L	10	10	0	100	V	III	Responder
TN18	F	79	L	2	10	0	100	IV	II	Responder
TN19	M	62	R	20	9	0	100	V	I	Responder
TN20	M	38	R	2	8	0	100	IV	III	Responder
TN21	F	71	L	6	9	0	100	V	II	Responder
TN22	M	64	R	2	10	0	100	V	III	Responder
TN23	F	61	R	7	10	0	100	V	II	Responder
TN24	F	60	R	2	10	0	100	IV	II	Responder
TN25	F	69	R	16	10	0	100	IV	III	Responder
TN26	M	60	R	3	10	0	100	V	III	Responder
TN27	F	79	L	11	9	0	100	IV	I	Responder
TN28	M	74	R	3	8	2	75	V	III	Responder
TN29	F	67	L	3	10	0	100	V	I	Responder
TN30	M	32	L	2	10	0	100	IV	III	Responder
TN31	M	73	L	1	8	0	100	IV	I	Responder
TN32	F	49	L	3	10	0	100	V	I	Responder
TN33	F	83	L	2	9	0	100	IV	I	Responder
TN34	F	64	R	4	10	2	80	IV	III	Responder
TN35	F	81	R	30	9	0	100	IV	III	Responder
TN36	F	69	L	9	9	2	78	IV	III	Responder
TN37	M	54	L	5	10	0	100	V	I	Responder
TN38	M	62	L	2	10	0	100	V	III	Responder
TN39	F	76	R	3	10	0	100	II	I	Responder
TN40	F	61	R	6	10	1	90	V	I	Responder
TN41	M	83	L	10	8	2	75	IV	III	Responder
TN42	F	79	L	22	9	0	100	IV	II	Responder
TN43	M	69	L	10	10	0	100	IV	I	Responder
TN44	F	49	R	5	7	0	100	V	III	Responder
TN45	F	39	R	8	10	2	80	IV	III	Responder
TN46	F	63	R	12	10	0	100	V	I	Responder
TN47	F	39	R	12	10	0	100	IV	III	Responder
TN48	M	83	R	9	8	0	100	IV	I	Responder
TN49	F	66	R	3	10	0	100	V	I	Responder
TN50	F	50	R	10	10	0	100	V	I	Responder
TN51	F	61	L	3	9	2	77	IV	III	Responder
TN52	F	71	R	2.5	8	0	100	IV	I	Responder
TN53	M	64	L	2	9	0	100	V	I	Responder
TN54	M	59	R	7	8	0	100	IV	I	Responder
TN55	F	56	R	3	9	0	100	IV	I	Responder
TN56	M	59	R	5	10	0	100	V	I	Responder
TN57	F	71	L	2	10	0	100	V	III	Responder
TN58	M	65	L	4	7	0	100	IV	III	Responder
TN59	M	66	L	6	9	0	100	IV	III	Responder
TN60	F	87	L	4	9	0	100	IV	III	Responder
TN61	M	53	L	21	10	0	100	IV	I	Responder
TN62	F	72	L	4	9	0	100	III	I	Responder
TN63	F	39	L	2	10	2	80	IV	III	Responder
TN64	F	74	R	9	10	1	90	IV	III	Responder
TN65	F	76	R	8	7	1	86	V	II	Responder
TN66	M	82	R	26	8	0	100	IV	I	Responder
TN67	F	79	R	22	9	0	100	IV	III	Responder
TN68	F	68	R	2	10	0	100	V	I	Responder
TN69	F	45	R	2	9	0	100	V	I	Responder
TN70	F	68	L	4	9	0	100	IV	III	Responder
TN71	M	71	L	30	10	3	70	IV	III	Partial
TN72	M	78	R	8	10	4	60	IV	IV	Partial
TN73	M	79	L	10	10	3	70	V	III	Partial
TN74	F	66	L	7	10	4	60	V	IV	Partial
TN75	M	64	L	4	10	4	60	IV	IV	Partial
TN76	F	82	R	10	10	3	70	IV	III	Partial
TN77	F	65	R	30	10	3	70	V	III	Partial
TN78	F	71	R	1	10	4	60	V	IV	Partial
TN79	F	82	R	9	8	3	63	IV	III	Partial
TN80	M	82	R	10	10	5	50	V	IV	Partial
TN81	F	58	L	2	10	4	60	V	IV	Partial
TN82	F	78	R	22	8	4	50	IV	III	Partial
TN83	M	60	R	2.5	9	3	66	IV	IV	Partial
TN84	F	73	L	11	10	5	50	IV	II	Partial
TN85	M	61	L	1.5	10	5	50	IV	II	Partial
TN86	F	88	L	3	10	4	65	IV	III	Partial
TN87	M	40	R	9	9	4	56	IV	III	Partial
TN88	M	43	L	1	10	3	70	IV	III	Partial
TN89	F	52	R	5	10	4	60	V	III	Partial
TN90	F	87	L	11	7	3	57	IV	III	Partial
TN91	M	76	R	22	10	5	50	IV	III	Partial
TN92	F	76	R	1	6	2	67	III	II	Partial
TN93	F	47	R	4	10	7	30	V	IV	Non-Responder
TN94	M	74	R	3	10	7	30	IV	IV	Non-Responder
TN95	M	38	L	1	10	9	10	V	V	Non-Responder
TN96	M	59	R	12	10	8	20	V	IV	Non-Responder
TN97	F	68	L	1	9	6	33	IV	V	Non-Responder
TN98	F	50	L	2	7	5	29	V	IV	Non-Responder
TN99	F	72	R	3	9	8	11	V	V	Non-Responder
TN100	M	65	R	13	3	4	−33	III	IV	Non-Responder
TN101	F	61	R	7	9	9	0	III	IV	Non-Responder
TN102	F	72	L	7	9	8	11	V	IV	Non-Responder
TN103	F	82	L	1	9	10	−11	V	IV	Non-Responder
TN104	M	70	R	2	8	9	−12.5	IV	IV	Non-Responder
TN105	M	81	L	5	10	10	0	V	IV	Non-Responder
TN106	F	68	R	5	10	10	0	V	IV	Non-Responder
TN107	F	31	L	3	10	8	20	IV	IV	Non-Responder
TN108	F	67	L	4	10	6	40	IV	IV	Non-Responder
TN109	M	85	L	20	10	6	40	V	IV	Non-Responder
TN110	F	80	R	3	7	4	43	IV	II	Non-Responder
TN111	F	59	R	4	10	6	40	V	IV	Non-Responder
TN112	F	46	R	6	10	8	20	IV	IV	Non-Responder
TN113	M	66	L	10	10	7	30	V	IV	Non-Responder
TN114	F	58	R	3	10	10	0	V	V	Non-Responder
TN115	F	35	R	2	6	10	−67	IV	V	Non-Responder

**Table A2:** *p*-values of cortical thickness change from pre- to post-surgery stratified by sex and response.

	Responder		Partial Responder		Non-Responder	
Regional Predictor	Female adj. *p*-Value	Male adj. *p*-Value	Female adj. *p*-Value	Male adj. *p*-Value	Female adj. *p*-Value	Male adj. *p*-Value
Contralateral:						
Isthmus cingulate gyrus	0.003	0.022	0.109	0.160	0.022	0.844
Fusiform gyrus	0.001	0.004	0.266	0.048	0.303	0.312
Pars orbitalis	0.272	0.410	0.519	0.769	0.083	0.641
Lateral orbitofrontal gyrus	0.820	0.791	0.733	0.492	0.276	0.547
Superior temporal gyrus	0.0001	0.001	0.064	0.232	0.003	0.148
Lateral occipital gyrus	0.820	0.615	0.733	0.845	0.803	0.742
Entorhinal cortex	0.0003	0.015	0.077	0.492	0.804	0.039
Ipsilateral:						
Fusiform gyrus	0.0004	0.003	0.469	0.232	0.151	0.461
Inferior temporal gyrus	8.73 × 10^−5^	0.006	0.424	0.193	0.012	0.148
Insula	0.448	0.263	0.423	0.556	0.761	0.843
Superior parietal gyrus	0.004	0.002	0.109	0.160	0.151	0.054
Pars triangularis	0.516	0.338	0.339	0.695	0.524	0.843
Lateral occipital gyrus	0.983	0.037	0.129	0.130	0.252	0.250

**Table A3:** Pre- and post-surgical cortical thickness in responders.

Responders			
Region	Avg. Pre-Sx Cortical Thickness (mm)	Avg. Post-Sx Cortical Thickness (mm)	Change in Cortical Thickness (mm)
Contra ICG	2.254779953	2.318005859	0.06322591
Contra FG	2.631626329	2.695561138	0.06393481
Contra POR	2.609807254	2.622457763	0.01265051
Contra LOFG	2.529764338	2.526269255	−0.0034951
Contra STG	2.520293568	2.605614319	0.08532075
Contra LOG	2.175712801	2.167097035	−0.0086158
Contra EC	2.968131851	3.195997166	0.22786532
Ipsi FG	2.627723732	2.701715211	0.07399148
Ipsi ITG	2.694906276	2.780237672	0.0853314
Ipsi Insula	2.824770092	2.851706754	0.02693666
Ipsi SPG	2.250938478	2.185734318	−0.0652042
Ipsi PTR	2.365778641	2.36605884	0.0002802
Ipsi LOG	2.184002078	2.175650785	−0.0083513

Average pre-, post-surgical, and change in cortical thickness for responders. Yellow boxes indicate statistically significant change from pre- to post- surgery with *p*-value < 0.05.

**Table A4:** Pre- and post-surgical cortical thickness in partial responders.

Partial Responders			
Region	Avg. Pre-Sx Cortical Thickness (mm)	Avg. Post-Sx Cortical Thickness (mm)	Change in Cortical Thickness (mm)
Contra ICG	2.281788307	2.338234682	0.05644637
Contra FG	2.622462306	2.686925708	0.0644634
Contra POR	2.555077912	2.571578284	0.01650037
Contra LOFG	2.561999127	2.543774879	−0.0182242
Contra STG	2.501347114	2.577481763	0.07613465
Contra LOG	2.20279712	2.183918432	−0.0188787
Contra EC	3.065825251	3.183936859	0.11811161
Ipsi FG	2.643999132	2.701415496	0.05741636
Ipsi ITG	2.695584081	2.765733636	0.07014956
Ipsi Insula	2.870699123	2.837415664	−0.0332835
Ipsi SPG	2.2510961	2.181719205	−0.0693769
Ipsi PTR	2.368328567	2.342690859	−0.0256377
Ipsi LOG	2.217537939	2.159627775	−0.0579102

Average pre-, post-surgical, and change in cortical thickness for partial responders. Yellow boxes indicate statistically significant change from pre- to post- surgery with *p*-value < 0.05.

**Table A5:** Pre- and post-surgical cortical thickness in non-responders.

Non-Responders			
Region	Avg. Pre-Sx Cortical Thickness (mm)	Avg. Post-Sx Cortical Thickness (mm)	Change in Cortical Thickness (mm)
Contra ICG	2.29462469	2.347016673	0.05239198
Contra FG	2.632255891	2.687430323	0.05517443
Contra POR	2.654746857	2.618848091	−0.0358988
Contra LOFG	2.559059637	2.599585923	0.04052629
Contra STG	2.528366009	2.633107796	0.10474179
Contra LOG	2.185175715	2.186783482	0.00160777
Contra EC	2.884453874	3.039644605	0.15519073
Ipsi FG	2.63196124	2.683129362	0.05116812
Ipsi ITG	2.685777381	2.757136334	0.07135895
Ipsi Insula	2.847255533	2.865932009	0.01867648
Ipsi SPG	2.255404051	2.18942624	−0.0659778
Ipsi PTR	2.363463516	2.367288348	0.00382483
Ipsi LOG	2.210340475	2.183689919	−0.0266506

Average pre-, post-surgical, and change in cortical thickness for non-responders. Yellow boxes indicate statistically significant change from pre- to post- surgery with *p*-value < 0.05.
